# Sustainable surgical resource initiative for Haiti: the SSRI-Haiti project

**DOI:** 10.1080/16549716.2023.2180867

**Published:** 2023-03-01

**Authors:** Richard Frechette, Nathalie Colas, Marc Augustin, Nathalie Edema, Gerson Pyram, Stanley Louis, Carl Eric Crevecoeur, Carmeline Mathurin, Raphael Louigne, Bhavesh Patel, Mitchell Humphreys, Alyssa Chapital, Mallory Martin, Qamarissa Ayoub, Daniel Hottinger, Michael T. McCurdy, Quincy Tran, Richard Skupski, Donald Zimmer, Mark Walsh, the SSRI-Haiti Project Group Contributors*

**Affiliations:** a Departments of Critical Care Medicine and Surgery, Saint Luke’s Medical Center, Port-au-Prince, Haiti; b Departments of Critical Care Medicine, Urology and Surgery, Mayo Clinic and Global, Surgical, Destination, Healthcare Inc., Phoenix, AZ, USA; c Bamiyan Maternal and Child Health Project and the Andeshgah Library, Kabul, Afghanistan; d Department of Anesthesia, Metropolitan Anesthesia Network, LLP, Plymouth, MN, USA; e Division of Pulmonary & Critical Care Medicine, University of Maryland School of Medicine, Baltimore, MD, USA; f Department of Medical Education, University of Indiana School of Medicine, South Bend/Notre Dame Campus, South Bend, IN, USA; g Department of Anesthesia, Memorial Hospital Beacon Medical Group of South Bend, South Bend, IN, USA; h Department of Emergency Medicine, Memorial Hospital Beacon Medical Group of South Bend, South Bend, IN, USA; i Departments of Emergency and Internal Medicine, Saint Joseph Regional Medical Center, Mishawaka, IN, USA

**Keywords:** Surgical procedures, Haiti, medical missions, general anesthesia, global surgical training

## Abstract

In response to the 2010 earthquake and subsequent cholera epidemic, St Luke’s Medical Center was established in Port-au-Prince, Haiti. Here, we describe its inception and evolution to include an intensive care unit and two operating rooms, as well as the staffing, training and experiential learning activities, which helped St Luke’s become a sustainable surgical resource. We describe a three-phase model for establishing a sustainable surgical centre in Haiti (build facility and acquire equipment; train staff and perform surgeries; provide continued education and expansion including regular specialist trips) and we report a progressive increase in the number and complexity of cases performed by all-Haitian staff from 2012 to 2022. The results are generalised in the context of the ‘delay framework’ to global health along with a discussion of the application of this three-phase model to resource-limited environments. We conclude with a brief description of the formation of a remote surgical centre in Port-Salut, an unforeseen benefit of local competence and independence. Establishing sustainable and collaborative surgery centres operated by local staff accelerates the ability of resource-limited countries to meet high surgical burdens.

## Background

Recent work has noted the low number of surgeons and anaesthetists as a major barrier for surgical patients in Haiti. Furthermore, most of the healthcare providers reside in the urban areas making it difficult for patients in rural areas to have access to surgical care [1]. The large volume of unmet surgical need in resource-limited countries has been the subject of enduring international attention [2–5]. Some indicate this volume is increasing as part of the ‘epidemiological transition’, in which the surgical burden of non-communicable diseases and injuries associated with increased life expectancy and demographic changes outpaces the concomitant increases in medical staff, facilities, education and mobility associated with socioeconomic development [5–7]. A recent prioritising of barriers to equitable surgical care in South Africa within the often-applied ‘delay framework’ (in this case, the *four* delay framework: seeking care, reaching care, receiving care and remaining in care) identified the following key barriers: provider education/training, surgical outreach, continuity of care, medical infrastructure, inadequate referral system, long waiting times and equipment capability and maintenance, seen in Table 1 [8]. As will be discussed below, we have created a three phase plan (build facility and acquire equipment; train staff and perform surgeries; provide continued education and expansion including regular specialist trips) for establishing and operating a sustainable surgical centre in a resource-limited environment; this straightforward plan addresses the aforementioned key barriers as well as surgical care within the broader delay framework of global health [8–12].

**Table 1.** Reproduced from [8]. Key barriers to improved access and quality of surgical care in South Africa.

Priority	Barrier
1	Lack of service provider’s knowledge, training and experience
2	Limited surgical outreach
3	Lack of and poor maintenance of equipment
4	Lack of surgical health education
5	Lack of decentralised services
6	Lack of continuity of care
7	Long waiting times
8	Complex and disjointed referral system
9	Problems with cost, time, safety, distance and comfort of transport
10	Lack of social support

Multiple strategies have been proposed to alleviate the surgical burden in low- and middle-income countries (LMICs), with some evidence suggesting that safe surgery is possible even in the most resource-scarce situations [13,14] and that surgical service trips can be cost-efficient according to World Health Organization standards [15,16]. Additionally, quality surgical care can raise international awareness to further attract resources to the areas of greatest need [17,18].

Increasing healthcare access and quality in LMICs is of intense global interest [5,19,20], yet the best methodology for delivering aid and resources remains uncertain [13–16]. Haiti struggles with a large volume of unmet surgical procedures despite robust regional and international support to improve care [21–23]. Its ability to provide surgical services is hampered by the lack of medical infrastructure, recurrent natural disasters, political upheaval and a recent escalation of local and international kidnappings and gang violence [24].

Healthcare in Haiti is delivered in one of the three manners: non-governmental organisations (NGOs), private hospitals with high costs only affordable to wealthy citizens, and private or public hospitals with low costs but which require patients to finance many of their own supplies and tests [20]. This healthcare network was strained beyond its limits during and after the 2010 earthquake and subsequent cholera outbreak [25]. These crises prompted an influx of medical supplies from the international community. During this period, a temporary medical centre named St Luke’s Hospital was constructed in Port-au-Prince.

St Luke’s was later made permanent and expanded to include a freestanding intensive care unit (ICU) and two operating rooms (ORs) with associated pre- and postoperative care areas. Three NGOs assisted in the initiation and support of these new surgical services at St Luke’s. The first was a group of general surgeons, anaesthesiologists, emergency physicians (EPs), and nurses from South Bend, Indiana, in association with South Bend Memorial Hospital and the University of Notre Dame. They first visited Haiti in August of 2012, when they began to construct an ICU and train local healthcare professionals in pre- and postoperative care. They also offered first-assist support to the local surgeons and provided training to enhance their medical decision making.

The second NGO to operate at St Luke’s was an orthopaedic group from Scranton, Pennsylvania, that began general anaesthesia cases in 2013. This group returns yearly for more complex orthopaedic cases and continued training of Haitian physicians and OR staff.

The third group is named Global, Surgical, Destinations, Healthcare Inc. (GSD) out of Mayo Clinic in Phoenix, Arizona. This group is a fully operational, self-funded team of urologists, general surgeons and anaesthesiologists along with preoperative, postoperative, circulating and first-assist nurses from Phoenix, South Bend and other medical centres with the important addition of critical care and emergency medicine physicians from the University of Maryland. This group also included anaesthesia assistance from anaesthesiologists and EPs from South Bend Memorial Hospital and Notre Dame. These groups returned annually and, in some cases, 3–4 times a year for ongoing support, complex cases and continuing education.

Local year-round Haitian staffing for the outpatient surgery unit consisted of two to three general surgeons, one anaesthesiologist, two scrub nurses, three to four preoperative and postoperative nurses, no technicians, and one to two autoclaving and cleaning crew members. After the 2019 departure of a nearby trauma centre run by a humanitarian NGO, two Haitian orthopaedic surgeons joined the team. Employment is consistent and there has been little turnover since the inception of the outpatient surgery clinic. Regarding foreign physician involvement in this program, there have been five general surgeons, three urologists, two EPs, two anaesthesiologists, three critical care physicians, ten family physicians, ten nurses and one physician assistant who have regularly participated in these trips and helped establish the surgery centre. Of these, there are two EPs, two critical care physicians, two anaesthesiologists, one urologist, three general surgeons with two scrub nurses and seven nurses who have consistently participated in multiple trips spread out over the year. Subsequently, other groups that offered speciality services performed more complex cases, such as mastectomies, maxillofacial surgery, complicated hernias, open laparotomies and orthopaedic surgeries.

Though these NGOs are foreign, they attempt to adapt to the existing Haitian medical environment as much as possible to improve that infrastructure and increase local medical staff competency via training and experiential learning. The funding for procedures continues to fall into one of the three categories: pro bono work, which constitutes all the surgeries performed by foreign physicians; means-tested repayment (for procedures performed by all-Haitian staff), which is a method instituted by St Luke’s to have patients pay for their medical services on a sliding scale based on income; or cash pay (also for procedures performed by all-Haitian staff) for those with the resources to pay for the full cost of treatment. In Haiti, the cost is a major obstacle to obtaining surgeries, but a patient in need is not denied care [1].

In this description, we begin with the acquisition and recruitment of equipment and staff for the surgical unit, followed by a description of staff training and types of surgeries performed and anaesthesia used. We then describe the continued relationship between volunteer medical professionals and local staff, which facilitated Haitian physicians and nurses assuming total control of the critical care and surgical units, and we delineate the education and expansion activities maintaining this independence and sustainability. We provide evidence of the increased number of complex surgical cases and use of general anaesthesia from the fall of 2012 until the fall of 2017 with comment on the 2018–2022 all Haitian staff cases. Finally, we discuss the work described here in the context of medical service in LMICs, offering thoughts on training and education, resource allocation/prioritisation and generalised vs. specialised procedures.

## Description

We describe the development of a sustainable surgery program in an LMIC environment, building on the literature regarding similar programs and key priorities in their implementation [8–12]. This three-phase plan is described below.

### Establish surgical rooms and equipment

As mentioned above, a temporary medical centre was established in 2010 following the earthquake and cholera pandemic and later expanded to include a freestanding ICU with two ORs. Crucial acquisitions and donations included two fully functional Dräger anaesthetic devices, two hospital operating beds, and air conditioning units that helped provide a sterile work environment such that one anaesthetist or anaesthesiologist and one assistant (usually an EP) could run two rooms simultaneously. Building sustainable facilities in resource-limited environments can require opportunistic acquisition of equipment no longer used by previous volunteers, as was the case for the Dräger devices and operating beds, left behind after the initial influx of resources in 2010. A technical advisor was secured to service all medical equipment and machines. Other ancillary surgical and medical equipment was donated by the many teams that visited. Noteworthy for the operation of this procedure was a fully operational autoclave machine that was donated during the cholera pandemic. With equipped ORs and ICUs in place, surgeries in this expansion to St Luke’s commenced, and staff training – already underway via local involvement in OR/ICU construction and equipment acquisition and setup – was accelerated as described below.

### Train for and perform surgeries

The initial surgeries were performed in one room by Haitian surgeons and anaesthetists/anaesthesiologists who performed simple genitourinary surgeries under spinal anaesthesia beginning in the late summer of 2012. Within months, foreign surgeons, assistants and scrub nurses began doing similar surgeries also with spinal anaesthesia. Soon Haitian surgeons, anaesthetists, anaesthesiologists, scrub nurses, and pre- and postoperative care nurses assumed more of the surgical load. Training was supplied by foreign – as well as Haitian – nurses and physicians. Within the first year, all-Haitian teams began to perform more complex surgeries using spinal anaesthesia independently. OR nursing/anaesthesia training also was provided by staff from St Damien’s paediatric hospital, a mature facility affiliated with St Luke’s. In April 2013, the first general anaesthetic cases were performed by teams predominantly from the United States. From that time until the present, there has been a gradual increase in overall surgeries performed, along with an increase in the complexity of cases (e.g. abdominal surgery and mastectomies) under general anaesthesia by all-Haitian teams as described in “Results” section.

Preoperative surgical recruitment and screening was provided by Haitian internists at the hospital-based clinic and at off-campus associated clinics (for those unable to reach the hospital easily) in order to reduce the barrier to care imposed by transportation. Appropriate postoperative management instruction was provided to local physicians and staff, which resulted in a sustained postoperative clinic managed by local physicians. Additionally, interaction between the hospital and surgical unit occurred for surgical patients requiring overnight observation due to complexity or presurgical morbidity, which rendered them unfit for discharge on the day of surgery. This focus on pre- and postoperative care and integration with the main hospital provided opportunity for experiential training and ultimately shorter wait times and improved continuity of care.

### Provide continued education and expansion including regular specialist trips

**Figure 1.**
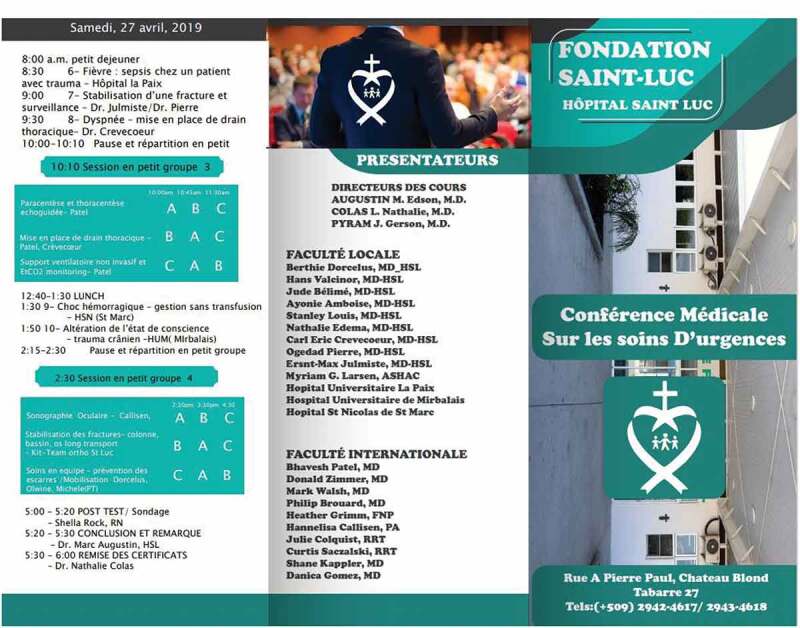
Brochure and schedule for 2019 National Medical Conference on Critical Care Conference organized and hosted by Haitian physicians and staff at St. Luke’s Hospital in Port- au-Prince.

Via the creation, staffing, training, and early success of the surgery centre, a cadre of Haitian internists came to serve both as critical care physicians and a local node of invaluable experience. This led to collaborative publications regarding critical care and anaesthesia in Haiti as well as a yearly interprofessional nationwide conference at St Luke’s Hospital in Port-au- Prince that was largely taught by Haitian faculty (Figure 1) [26–37]. These lectures are attended by nurses and physicians throughout the entire region. Continued education for providers in Haiti became important because of the withdrawal of the international NGO trauma team in the fall of 2019 which had served the area around St Luke’s Hospital. As a result, many of the local trauma patients arrived at the hospital and required treatment of fractures and surgery as well. The educational program addresses this and other concerns regarding care of trauma patients. Initial funds for the establishment of this program were provided by USAID to build a simulation centre, a grant from the Mayo Clinic and donated simulation equipment from GSD Healthcare.

These combined efforts have culminated in a surgical centre with local healthcare professionals who address the most prevalent surgical needs of the region independently. This has allowed for targeted, ‘vertical’ trips by specialists to donate their expertise in short bursts to handle the caseload of the less commonly occurring surgical procedures. Vertical medical mission programs refer to foreign-based surgical teams that perform advanced surgical procedures, such as the repair of cleft-lip and palate deformities during specific periods [38]. The Notre Dame group had specific nonsurgical experience in Haiti for decades as well as the foreknowledge of such ‘vertical’ ophthalmological surgery trips to Madagascar [39]. We benefited from such vertical integration with the biannual urological evaluations and performance of holmium laser enucleation of the prostate (HoLEP) for previously screened patients by GSD of Phoenix, Arizona. During these visits, local anaesthesiologists often assisted in the performance of other urologic cases. Local nursing staff performed crucial preoperative screening and provided postoperative care, while local surgeons also received training and experience on these mission-oriented trips. Equipment cleaning, sterilisation and room turnover were performed by local surgical technicians.

Special attention was taken to deliver surgical care in accordance with the U.S. standards of care for all patients. This included established best practices, Health Insurance Portability and Accountability Act (HIPAA) protections for all patients, and informed consent. When materials, laboratory studies, or radiologic studies were unavailable, the foreign and local teams worked together to adapt and improvise using resources available locally. Additionally, an independent review board based in the U.S. reviewed all cases. Direct oversight was provided locally by the board of St Luke’s Hospital including Dr Fr Richard Frechette, CP.

With this sustainability-focused three-phase plan described, we next share the description and evolution of procedures performed and types of anaesthesia employed by both foreign and Haitian physicians.

## Application of the three phase model to establish a sustainable surgical center in Haiti

**Figure 2.**
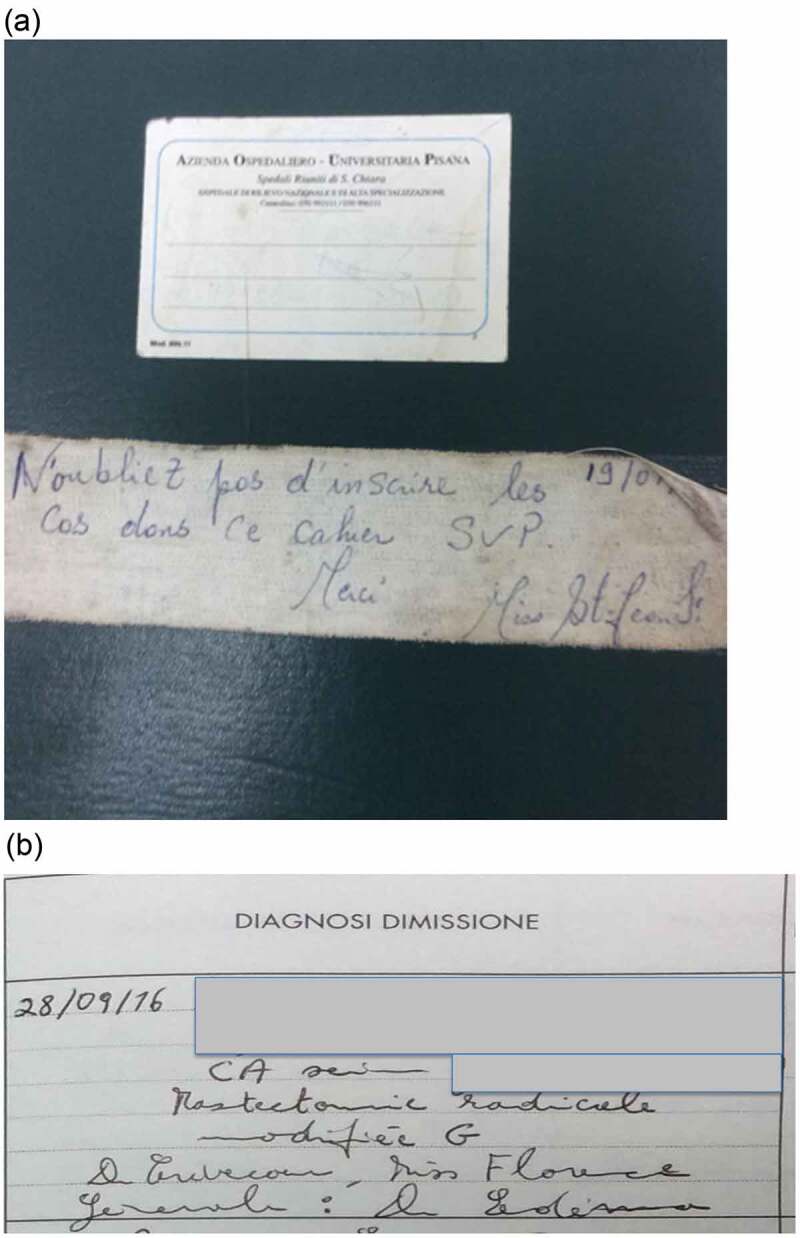
(a) Surgery log book arrived after the earthquake provided by an Italian team. (b) Example of an entry from the logbook detailing the date, patient’s name, age, medical record number, diagnosis, name of surgeon and assistant, type of anesthesia and anesthetist/anesthesiologist. For this patient on October 28th, 2016, with a breast cancer had a modified radical mastectomy with surgeon Dr Crevecouer, assisted by nurse Miss Florence with general anesthesia by Dr Edema.

At the end of each case, the patient’s name, date, anaesthesia used, provider of anaesthesia, surgeon, and assistant (if present) were listed in a logbook kept in the surgical unit; a picture of the cover of the log book and an example case entry from the logbook is shown in Figures 2a,b. It was the duty of the anaesthesiologist or nurse anaesthetist to record each case in the surgical log. A limitation of this documentation is the incomplete or illegible entrance of data within the logbook, which excluded a small percentage of the cases. The types of surgery performed by both Haitian and foreign physicians are listed in Table 2.

Translation: “Do not forget to write down the cases in this notebook. SVP name of nurse”. This obligation was strictly enforced by the nursing director of the surgical centre and performed by the anaesthetist or anaesthesiologist.

**Table 2.** Types of cases performed, Aug 2012–Dec 2017 inclusive. Note that there were 1,651 known procedures performed between August 2012 and December 2018 inclusive. However, following the end of December 2017, foreign intervention shifted focus from providing surgical and anaesthesia support to critical care education. Therefore, we have excluded 145 cases, which occurred between January 2018 and December 2018, meaning analysis was only performed on the 1,506 cases from 2012 to 2017. In addition, participation in surgery, anaesthesia and education as well as data collection and analysis were hindered starting in mid-2019 and early-2020 because of national political upheaval and gang violence and travel restrictions caused by the COVID-19 pandemic, which together precluded continued participation in and analysis of the surgical program [40–43]. As of the writing of this paper, St Luke’s continues to perform surgeries with spinal, local, and general anaesthesia independently. Table 2 was composed from a descriptive statistical analysis on cases performed between Aug 2012 and Dec 2017.

Surgery type	Foreign physicians	Haitian physicians	Total
Circumcision	73 (4.8%)	36 (2.4%)	**109 (7.2%)**
Excision	69 (4.6%)	236 (15.7%)	**305 (20.3%)**
Hemorrhoidectomy	4 (0.3%)	25 (1.7%)	**29 (1.9%)**
Hernia	130 (8.6%)	379 (25.2%)	**509 (33.8%)**
Holmium laser enucleation	83 (5.5%)	0 (0%)	**83 (5.5%)**
Hydrocelectomy	63 (4.2%)	192 (12.7%)	**255 (16.9%)**
Mastectomy	19 (1.3%)	42 (2.8%)	**61 (4.1%)**
Miscellaneous	42 (2.8%)	27 (1.8%)	**69 (4.6%)**
Multiple surgeries	20 (1.3%)	66 (4.4%)	**86 (5.7%)**
**Total**	**503 (33.4%)**	**1003 (66.6%)**	**1506 (100%)**

**Figure 3.**
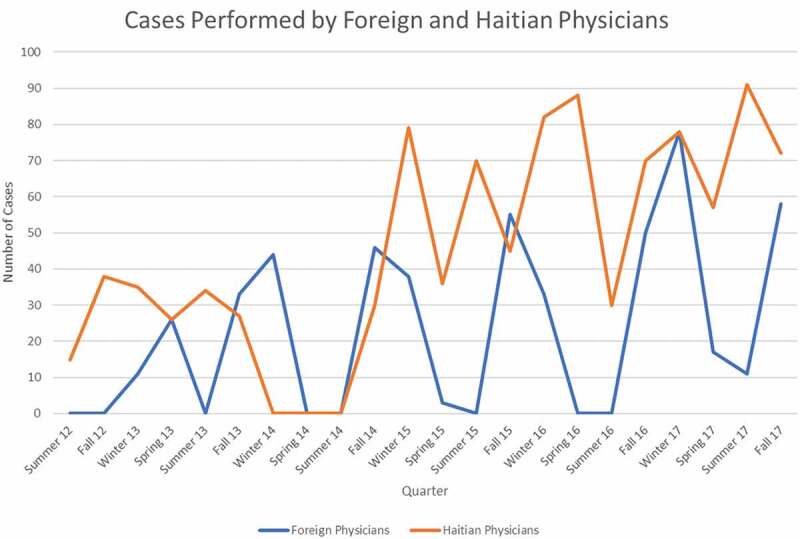
Total number of cases at St Luke’s Hospital in Port-au-Prince on a quarterly basis, differentiated between Haitian doctors and foreign doctors.

Table 2 reveals certain trends, as follows: the comparison of foreign and Haitian surgeons and anaesthesiologists reveals a preponderance of circumcisions done by foreign physicians. The reason for this difference is that hospital employees who worked for the surgical unit used this opportunity to obtain circumcision as part of a program to reduce the transmission of HIV, which has been shown to be reduced by circumcision by as much as 60% [44]. The other major difference is the HoLEP surgeries done only by Mayo Clinic urologists as part of a vertical surgical program funded by GSD of Phoenix, Arizona and Lumenis, the company that manufactures the HoLEP. This machine is now stored at the hospital, and a technical advisor provided by Lumenis accompanies GSD on its biannual visitation to St Luke’s Hospital. Figure 3 describes the total number of cases (shown in Table 2) performed by the Haitian and foreign physicians between 29 August 2012 and 21 December 2017. The number of cases performed by Haitian surgeons clearly increased over time, never exceeding 40 per season in the first 2.5 years and exceeding 40 cases for all but two seasons during the final 3 years plotted. Additionally, the yearly maximum number of cases per season increased within the final 3 years: 79 in 2015; 88 in 2016; and 92 in 2017. There is also a slight upward trend in the yearly cases performed by foreign surgeons, seen easily due to the seasonal nature of the foreign contribution.

**Figure 4.**
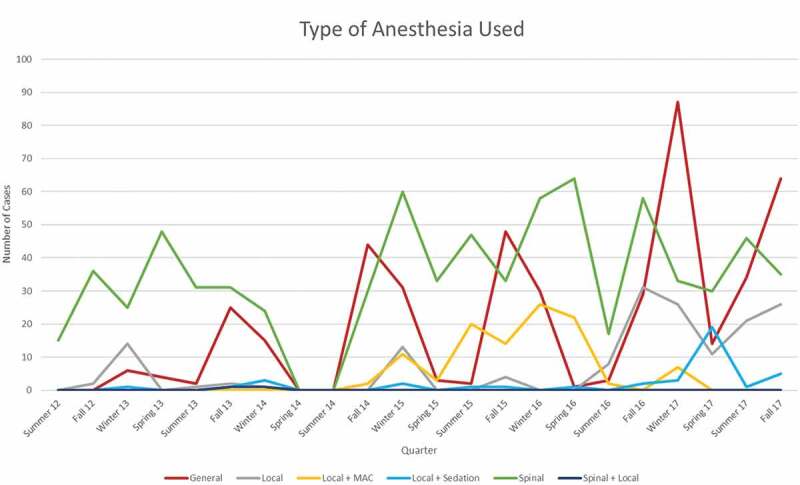
Total number cases at St Luke’s Hospital on a quarterly basis. It is differentiated by the type of anesthesia applied. There are six categories of anesthesia which includes general, local, local + MAC, local + sedation, spinal, and spinal + local.

The type of anaesthesia evolved from the initial use of spinal anaesthesia by both the Haitian and foreign physicians to a gradual adoption of general anaesthesia for specific cases, as seen in Figure 4. It is worth noting that concomitant with this increase in case complexity, the auxiliary roles of nursing and in-hospital care for patients who required overnight observation were increasingly filled by Haitian nurses and critical care physicians, both of whom were trained by foreign physicians and nurses.

**Figure 5.**
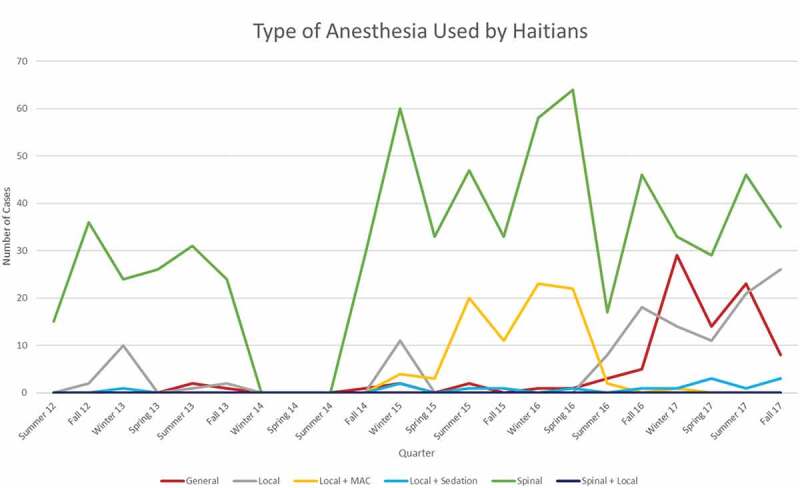
Types of anesthesia used by all-Haitian teams on a quarterly basis. It is differentiated by the type of anesthesia applied. There are six categories of anesthesia which includes general, local, local + MAC, local + sedation, spinal, and spinal + local.

At the beginning of this period of analysis, Haitian nationals performed at best 30–40 procedures per quarter with many quarters having little to no surgical cases. With each targeted and relationship-building visit from foreign medical professionals, the local medical staff improved their ability to perform and maintain surgical services in the absence of outside assistance. Within 6 years of the start of this project, the local Haitian healthcare team consistently performed over 2.5 times the number of procedures they previously performed sporadically. Likewise, the diversity and complexity of the Haitian-performed caseload increased, as seen in Figure 5. Most noticeably in Figure 5 is the change occurring in the fall of 2016, when there was an increase in the number of general anaesthesia cases performed by Haitian staff. This was due to the fact that the surgery centre and hospital had recruited their own Haitian surgeon who on his first day on 30 August 2016, for example, did three mastectomies under general anaesthesia. A sustained increase in local- and local-plus-anaesthesia cases performed in aggregate and by Haitian staff began in the winter of 2015 (Figures 4 and 5), representing a broadening of treatment capability at St Luke’s. Investing time and resources by both foreign and Haitian practitioners yielded a clear increase in the capability of local and combined teams to perform complex and more varied cases.

Some of the more complex cases included radical and modified mastectomies, large soft tissue masses, maxillofacial surgery, complicated hernias, open laparotomies, orthopaedic surgeries, and incision and drainage of Fournier’s gangrene and infected hydrocele. As of 2023, Haitian physicians perform more complex procedures, such as open abdominal surgeries using general anaesthesia.

A concerted effort was made to only schedule cases with low risk of complication unless the case for intervention overwhelmed the risk for complication. There were no deaths related to surgery or anaesthesia in the intraoperative or post-operative periods.

## Discussion

This work describing the creation and evolution of a single surgical unit adds to the large body of the literature on medical care and surgery in LMIC environments in general and Haiti specifically, which includes discussions of facility funding, barriers to effective care and shortcomings of current volunteer programs, comments on how to improve access to care, surveys of multiple facilities, and a combined description of previous cases at St Luke’s and a similar institution in Honduras [2,5,8,10,12,19,21,23,26–38,45,46]. In the existing literature, there are similar examples of scaling up of surgical care in rural Haiti, which has been described by the Partners in Health and the Zanmi Lasante (PIH/ZL) organisations. This resulted in the construction of surgical suites at three hospitals and clinics serving approximately 3.3 million people [1]. Establishing surgical care in resource-limited nations continues to represent a difficult and multifaceted effort combining acquisition and maintenance of medical infrastructure and equipment, training and recruiting local and foreign physicians, and sustaining long-term high-quality performance [47–49]. We have demonstrated that a three-phase plan, detailed earlier, can address these simultaneous challenges, and here we briefly discuss our plan in the context of the so-called delay framework (or delay model) to global health, which owes its origin to a maternal mortality program in sub-Saharan Africa [8–12]. Our three-phase plan, which led to a sustainable surgical centre in Haiti, addresses the main barriers to surgical care recently identified with the four delay framework, apparent by considering the description herein compared with barrier prioritisation in Table 1 [8]. Note for barrier identification, the four delay framework (seeking care, reaching care, receiving care, and remaining in care) is equivalent to its predecessor, the three delay framework which originated as part of maternal mortality studies (its three delays being seeking care, reaching care and receiving care). The four delay framework divides the ‘receiving care’ delay among two separate delays: receiving care and remaining in care [8,9]. More broadly, the work described here addresses the important delays identified across a variety of care types in LMICs via the delay framework, as seen in Table 3.

**Table 3.** The SSRI three phase plan within the context of the delay framework. Each delay in the four delay framework is listed in the left column; each phase in the 3-phase plan is listed on the top row. The entries in the body of the table indicate how each phase addresses each delay.

	Phase 1 Build facility and acquire equipment	Phase 2 Train staff and perform surgeries	Phase 3 Continued Education and Expansion
Delay 1 Seeking Care	Perform proactive outreach with community leaders and medical staff	Delivery of quality care combined with local outreach encourages care-seeking by local population and “word-of-mouth” communication	Haitian-led conferences with foreign contributors; NGO peers and local churches/leaders continue spreading word.
Delay 2 Reaching Care	Creation of local satellite clinics for screening; Remote outpost in Port-Salut made possible by local competence in Port-au-Prince and volunteer trips	Deploy referral and preoperative screening protocols; Continued engagement with churches and local leaders for patient transport and awareness	Continued experiential, classroom and conference training on referral and preoperative screening protocols
Delay 3 Receiving Care	Maintain facility/equipment and continue acquisition as needed	Deploy operative protocols; Train local physicians on surgical practices; Perform surgical care with local and combined staff	Help ensure local sustainability while continuing seasonal targeted visits for volunteer care; Continued education on operative and maintenance protocols
Delay 4 Remaining In Care	See above (maintain facility/equipment)	Deploy follow-up care protocols and patient education regarding importance of follow-up and early care-seeking in the event of symptoms	Continued staff education on follow-up protocols

As Table 3 shows, the SSRI program addresses the four delay framework in multiple ways. Expanding on the discussion in the table, we suggest that establishing a surgical facility in an area of demonstrated need addresses the first and second delays, as providing a source of surgical care in a high-population area helps people to reach the hospital and incentivizes people to seek care because of low costs and readily available surgeons and anesthetists/anesthesiologists. By training staff to expedite the workflow of the facility, patients receive surgical care without delays, facilitating patient engagement in recovery and follow-up, thus addressing the third and fourth delays. Recent studies have found that nations with poor access to resources have more postoperative deaths, especially due to avoidable issues such as surgical infections, compared to nations with more resources [50], and the success of our program is due in part to careful pre-screening and post-procedure practices. These practices include: pre-screening clinics staffed by physicians to identify patients for surgery with hospital-based coordination of transportation; the previously described implementation of our surgical program with hospital-based follow-up and recovery practices including in-hospital care; critical care for the sicker patients; and where possible follow-up with the same screening physicians who coordinated surgical referral [8]. With the experience and results at St Luke’s in Haiti described, we next share insights regarding the application of our three phase model to resource-limited areas in general.

### Delay #1: delays to seeking care after symptom onset

Most surgeries during the study period were elective operations to treat chronic hernias and hydrocoeles. Regarding the Holmium laser enucleations of the prostate (HoLEP) done by the Mayo team, each patient was evaluated in a clinic 6 months and then again 1 week prior to the surgery by local physicians. A majority of the patients had symptoms for months to years prior to surgery. Acute surgeries were done for acute abdominal pain or severe illnesses, such as Fournier’s gangrene or scrotal abscesses whereby the duration of symptoms was less than 1 week.

### Delay #2: number of patients screened at satellite clinics, patients presenting with late-stage disease [51]

Approximately 15–20% of patients had a greater than 2 h commute to St Luke’s in Tabarre, a suburb of Port-au-Prince, from Cité Soleil, which is a rendezvous point for the St Luke’s shuttle. Authors and contributors have established a satellite clinic in the dangerous centre of violence, Cité Soleil, which is notorious for gang violence and has served as a referral source for patients. Until 2018, the level of violence was significant but stable. However, since the social unrest and local gang warfare has roiled Haitian infrastructure in July of 2018, delays have become much greater and the volume of referrals from the referring clinic has dropped precipitously. Of note, most of these patients presented with late-stage disease as exemplified by the high number of patients who had large, complicated hernias.

### Delay #3: time to surgery after presentation

Since these surgeries were for the most part scheduled, once the patient arrived, usually in the morning, surgery was done the same day. For those patients whose surgery was not able to be done on the same day, they would spend the night in the pre-operative suite cared for by the night-nurse assigned by the hospital.

### Delay #4: loss to follow up

St Luke’s has a significant pre-operative screening and scheduling process using its extensive network of local Haitian healthcare providers and administrators. There are extensive records of the patients’ addresses, cell phone numbers, and family contacts. One of the strengths of this program is the long-standing affiliation of St Luke’s Hospital and the freestanding St Luke’s outpatient surgery centre to The St Luke Foundation for Haiti. As a result of this close-knit web of employees and medical practitioners, patient follow-up is nearly complete which reflects the interconnected nature of the Saint Luke Foundation community particularly in such a violent and chaotic environment as in Haiti of the last 5 years.

#### Generalized implementation in LMICs

Here, we describe application of the three-phase model to a resource-limited environment. A generalised application can be inferred from the description above of what was done in Haiti, and here we focus on the key and/or universal aspects of the plan that bears further discussion for generalised applications.

#### Establish surgical rooms and equipment

First, establish an acute care facility with sufficient power, water, and environmental control capabilities for treating surgical complications. Next, build a structural unit consisting of ORs with a pre- and postoperative unit attached. With those structures in place, obtain the remainder of the necessary supplies including telemetry, hospital beds, intravenous poles, etc.

These first preparatory steps are an obvious target for allocation of funds from international aid to resource-limited nations. The commitment of a technological advisor to the project is key as the donated materials will have a wide range of brands and functionality. Without support systems for these auxiliary systems in place, their dysfunction and eventual failure of operation is inevitable [8,52–54].

#### Train and perform surgeries

Local surgeons and anaesthetists/anaesthesiologists along with nursing and technician staff are collaboratively identified by local medical leaders and foreign physicians and staff for targeted training in preoperative screening and postoperative care with a special emphasis on airway management. These first individuals are used as anchors for the continued growth and development of peers within their individual area of training. Once that training is complete, the first surgeries are scheduled, selecting cases that involve simple spinal anaesthesia and local anaesthesia with moderate sedation. These first surgeries should be performed by local staff in all roles as much as possible. As all parties become comfortable with the workflow and process, the caseload is increased gradually and broadened to include general anaesthesia. This phase can be facilitated by planned visits from foreign professionals over several week periods.

#### Provide continued education and expansion including regular specialist trips

The next phase is to reinforce local staff capability via repetition/experience, continued education via conferences, training sessions, and traditional classroom and textbook readings, communication with associated foreign volunteers, and continued work with foreign staff during seasonal and/or specialised vertical mission trips as described previously. Note that conference topics, training sessions and classroom subjects include both surgical and non-surgical materials for physicians, providers of anaesthesia, nurses, and other medical staff. This consistent, experience- and relationship-based capability growth strategy facilitates the acquisition of generational knowledge within the local community, without which sustainability is impossible. Partnership with local leaders and stakeholders facilitate social acceptance and psychological safety, which is foundational to successful transfer of knowledge and calibration of expectations.

This model seeks to strengthen and build medical infrastructure, which is eventually maintained and operated almost exclusively by local staff. Rather than launching immediately into highly specialised procedures, this model enables the local professionals to manage a high volume of routine cases with a manageable amount of additional training. This system creates a foundation for future aid involving procedures in specialised areas to be scheduled and performed by foreign surgeons. Thus, the three-phase model combines the long-term infrastructure-and-training relationship necessary for a locally operated sustainable surgery centre with specialised vertical surgical missions, the latter meeting a medical need and offering further training and exposure to local staff as they continue building competency and generational knowledge.

Several previously mentioned items worthy of special attention include: specific and high-level instruction concerning pre- and postoperative care be given to the nurses; formation of an ICU, critical care team, and a series of recurring critical care conferences to ensure the continuity of high-quality care for the most at-risk patients during their time outside of the OR. Without these important facets of the model, negative outcomes are more likely to result than sustainable surgical care. For example, the foreign physicians might perform all the procedures without teaching pre- and postoperative care, and much of their added benefit is lost when they leave the location [22]. In the other scenario local surgeons and anaesthesiologists would be trained in more complex procedures, but a growing number of poor outcomes result due to poor screening, post-surgical complications, and poor support in the convalescing period [55]. In our implementation, the GSD practitioners were crucial in these aspects of the model. At each visit from the GSD team, the local workers, including the housekeeping staff, the nursing staff, and the surgeons and anaesthesiologists, received training to expand their expertise.

We propose this model as a rebuttal/alternative to the criticism of some short-term medical mission models that have been called ‘medical volunteerism’ [56]. As has been commented on in [38], some NGOs function through short-term medical missions, which perform a wide array of routine surgeries, which could be performed by local physicians. However, with this method often comes no exit strategy, and when they leave, much of the benefit they brought leaves with them [38]. It is our suggestion that a better model is to have a continued ‘relationship strategy’. By putting emphasis on training local staff on routine procedures and improving local medical infrastructure, we were able to create a self-sustainable local team that could continue performing these procedures after foreign physicians left. We also created a long-standing relationship in which we continue to return to Haiti to perform more specialised procedures and reinforce competencies. Our experience suggests that this strategy is a key to our model’s effectiveness.

Some obstacles still exist with this method. The local language is usually the first and largest barrier to this method, especially in the beginning. This often requires a commitment from at least one foreign physician to learn the language and take the lead in some of the early steps. Local translators are key to allowing the growth of the program in such a way that more foreign specialists can make trips into the area and share their expertise. Securing early resources for materials is another obstacle depending on the location and availability of international interest in funding healthcare improvements in an area. Notably, this model attempts to avoid the dysfunction that can result from starting a system that runs parallel to rather than within the existing medical infrastructure [49].

#### Consequences of increased local surgical demand and access

We conclude with an anecdote, which we believe demonstrates the cascading benefits of sustainability when integrated with continued – and, as needed, improvisational – collaboration with foreign staff. The number of foreign surgeons and anaesthesiologists reached a peak in the fall of 2017 and winter of 2018. Subsequent surgeries performed by foreign teams not analysed in this paper decreased significantly in 2018 and 2019 because of the full capacity of the surgery rooms, which were utilised by all-Haitian teams. As a result, a team of foreign physicians in 2019 was prepared to perform surgeries in Port-au-Prince but was unable to do so because of the scheduled procedures by local surgeons and anaesthesiologists. Therefore, the foreign team transported a universal anaesthetic machine and a team of anaesthesiologists and surgeons with family physicians and nurses who performed surgery at a recently acquired medical facility distant from Port-au-Prince. The result was that a small team of foreign physicians established a satellite surgery unit in an affiliated hospital in the rural town of Port-Salut, which is on the southwest-most tip of the Haitian peninsula. This begins to address the concern regarding the absence of surgical services in rural settings of LMICs and serves as an example demonstrating how sustainability can have cascading benefits: local independence at one location diverts foreign aid to a different location, which needs it more, leading to a satellite unit at the new location [38]. As mentioned at the beginning this report, two competing phenomena generally both increase with economic growth and life expectancy in an LMIC environment: 1) surgical cases, particularly those associated with injuries and non-communicable diseases, and 2) availability and quality of care to meet this increased surgical burden (i.e. hospital quality, number of physicians and nurses, practitioner/staff education). We submit that establishing sustainable and collaborative surgery centres operated by local staff accelerates the second phenomenon in ways expected and unexpected.

#### 2018–2022 all Haitian surgery, durable independence, meeting local need

To perform complete analysis, we ended our description in the late Fall of 2017. In depth collection and recording of subsequent data in 2018 and 2019 was limited by cancellation of several trips because of the political upheaval and the July 2021 assassination of the Haitian president, Jovenel Moïse. Haitian fuel protests over increased fuel prices which occurred in early July 2018 resulted in numerous protests throughout 2019, 2020, 2021, culminating in the assassination. In addition, the 2020 COVID-19 pandemic further eroded social order. This instability transiently limited services by St Luke’s outpatient surgery centre and unfortunately resulted in incomplete data for 2018 even though there was one foreign trips as late as January 2019 to Port-Salut. The murders, kidnapping and violent attacks on local Haitians employed by the hospital is an example of the chaos the satellite clinic and hospital have faced. For example, within the space of 10 days in July of 2022, more than 200 people died as gangs fought over the control of Cité Soleil. Many of those killed had no relation to gangs. Cité Soleil was until 2018, a vibrant satellite clinics affiliated with St Luke’s hospital. For these reasons, it was difficult to provide in depth data post-2018. However, local Haitian physician have kept track of the dates, types and number of cases performed since the departure of the foreign physicians. Surgical volume has increased due to the need to attend to the augmented volume of orthopaedic trauma. This required a retooling of the surgical facilities to accommodate the increased volume.

By comparison, some of the co-authors and contributors have assisted in the expansion of a very robust inpatient surgery program at Hopital Sacre-Couer, in the northern Haitian town of Milot which was also temporarily shut down due to COVID-19 and violence. This hospital is a so-called ‘Bellwether’ hospital as defined by the Lancet Commission on Global Surgery in that it is capable of caesarian sections, laparotomies, and the repair of open fractures [1]. This hospital has resumed offering surgical services with foreign physician participation for orthopaedics in order to assist in surgeries, which have been ongoing during this period due to the lessened violence in the northern rural town of Milot.

The last complete year of data analysis was for 2017. Retrieving the data from the log book and entering of cases into the main spreadsheet with subsequent evaluation was hampered by the social and political unrest which made travel to Haiti difficult. However, through local Haitian physicians, an up-to-date simplified and numerical review of the surgical caseloads reveals that, despite the intermittent interruption of delivery of surgical care caused by the July 2018 deterioration of social and political order further complicated by the 2020–2021 COVID-19 pandemic, the surgery centre continued to perform surgery by all Haitian teams at an even greater pace than before 2018.

Specifically, 516, 541, 554, 417, and 389, surgical cases were done in 2018, 2019, 2020, 2021 and 2022. However, most cases were orthopaedic. Due to the departure of the local humanitarian NGO supported trauma centre in 2019 caused by safety concerns in the Tabarre suburb of Port au Prince, Saint Luke’s Hospital hired two orthopaedic surgeons and used the surgical centre as their basis of operations as a substitute trauma centre. One of these surgeons previously worked at the orthopeadic speciality facility, Hopital Sacre Couer in the more rural North where violence is less and had extensive experience in trauma-related orthopaedic surgery. This orthopaedic surgeon and other coauthors of this paper and contributors to the development of the Saint Luke’s surgery centre had previously published his results concerning his work in Milot [35]. He and another orthopaedic colleague, with the support of Saint Luke’s Hospital, established a supply chain source for orthopaedic equipment from India, which allowed the patients and hospital to purchase lower cost orthopaedic hardware for lower extremity and hip fractures. The hospital also purchased a C arm fluoroscopy X-ray machine as well as an autoclave machine able to handle the sterilisation of equipment necessary for the increased cases of orthopaedic injuries. Local staff were cross trained as orthopeadic technicians, with the other staff assuming new duties related to the increase in orthopaedic trauma cases. As a result, the number of cases from 2019 increased significantly. Numeric analysis of the data since 2018 reveals that 60% of cases were orthopaedic cases, with the majority being related to trauma, while the remaining 40% of the cases were for elective herniorrhaphies and hydrocelectomies. By comparison, the greatest volume of cases from 2012 to 2017 was in 2016 when all Haitian surgeons and anaesthesiologists performed 374 surgeries. Of note is that the greater availability of operating time due to the absence of foreign physicians from 2018 allowed greater flexibility for the Haitian teams as evidenced by the need for the January 2019 all-foreign team to embark on a rural trip surgical trip to the rural southwestern peninsula town of Port-Salut because of a full all Haitian schedules. This collaboration demonstrated the utility of cross-fertilisation of both Haitian and foreign physicians working at diverse hospitals in Haiti utilising their shared experiences as well as the ability of the local Haitian administrators and staff to adjust to the increased demand for new challenges in orthopaedic trauma cases.

## Conclusion

The sustainable surgical resource initiative (SSRI) for Haiti provides a model for improved delivery of high-quality surgical healthcare while continually enhancing the skills of local surgeons, anaesthetists, anaesthesiologists, internists, and nurses. The three phase model (establish facility and equipment; train staff and perform surgeries; perform continued education and expansion including regular vertical specialist trips) led to a sustainable surgical center and a node of generational knowledge. We have documented an increase in number and complexity of cases performed by all-Haitian staff over a period of 6 years (2012–2017). Rather than engage in short-burst visits performing routine surgeries (which could be performed by local physicians) with unpredictable frequency and no exit strategy, we recommend a sustainability-focused and relationship-based strategy whereby foreign surgical units with the support of NGOs establish medical infrastructure for surgical intervention and offer training of local surgeons, anesthesiologists, and other auxiliary medical professionals. This allows for the local medical professionals to continue performing a high volume of cases independently. The SSRI model is applicable in addressing the four delay framework to global health, generalisable to the challenges of many resource-limited surgical environments, and can be adapted to the specifics of the language, culture, and existing local medical infrastructure.

## Paper context

Developing sustainable surgery centres in LMIC environments is crucial to the improvement of global health. Here we report the conception, staffing, training, procedures, data collection and follow-up for a sustainable surgery centre in Haiti and discuss the methods and results within the prevailing paradigm of global healthcare, the Delay Framework. We stress the relationship-based approach between local and foreign staff, noting the importance of prioritisation in resource allocation to ensure sustained local capability.
